# Human visual consciousness involves large scale cortical and subcortical networks independent of task report and eye movement activity

**DOI:** 10.1038/s41467-022-35117-4

**Published:** 2022-11-29

**Authors:** Sharif I. Kronemer, Mark Aksen, Julia Z. Ding, Jun Hwan Ryu, Qilong Xin, Zhaoxiong Ding, Jacob S. Prince, Hunki Kwon, Aya Khalaf, Sarit Forman, David S. Jin, Kevin Wang, Kaylie Chen, Claire Hu, Akshar Agarwal, Erik Saberski, Syed Mohammad Adil Wafa, Owen P. Morgan, Jia Wu, Kate L. Christison-Lagay, Nicholas Hasulak, Martha Morrell, Alexandra Urban, R. Todd Constable, Michael Pitts, R. Mark Richardson, Michael J. Crowley, Hal Blumenfeld

**Affiliations:** 1 Department of Neurology, Yale University, New Haven, CT USA; 2 Interdepartmental Neuroscience Program, Yale University, New Haven, CT USA; 3 Biomedical Engineering and Systems, Faculty of Engineering, Cairo University, Giza, Egypt; 4 Child Study Center, Yale University, New Haven, CT USA; 5 Division of Psychology and Language Sciences, University College London, London, UK; 6 NeuroPace, Inc., Mountain View, CA USA; 7 University of Pittsburgh, Pittsburgh, PA USA; 8 Department of Neurosurgery, Yale University, New Haven, CT USA; 9 Department of Radiology and Biomedical Imaging, Yale University, New Haven, CT USA; 10 Reed College, Portland, OR USA; 11 Department of Neurosurgery, Massachusetts General Hospital, Boston, MA USA; 12 Harvard Medical School, Boston, MA USA; 13 Department of Neuroscience, Yale University, New Haven, CT USA

**Keywords:** Consciousness, Perception, Sensory processing

## Abstract

The full neural circuits of conscious perception remain unknown. Using a visual perception task, we directly recorded a subcortical thalamic awareness potential (TAP). We also developed a unique paradigm to classify perceived versus not perceived stimuli using eye measurements to remove confounding signals related to reporting on conscious experiences. Using fMRI, we discovered three major brain networks driving conscious visual perception independent of report: first, increases in signal detection regions in visual, fusiform cortex, and frontal eye fields; and in arousal/salience networks involving midbrain, thalamus, nucleus accumbens, anterior cingulate, and anterior insula; second, increases in frontoparietal attention and executive control networks and in the cerebellum; finally, decreases in the default mode network. These results were largely maintained after excluding eye movement-based fMRI changes. Our findings provide evidence that the neurophysiology of consciousness is complex even without overt report, involving multiple cortical and subcortical networks overlapping in space and time.

## Abstract (web summary)

Isolating the neural mechanisms of consciousness is complicated by task report and other irrelevant signals. Here, the authors removed report and eye movement confounds to uncover large scale cortical-subcortical networks specific for human visual consciousness.

## Introduction

Consciousness is central to human experience yet is not easily explained. Recent work suggests that the full richness of conscious experience may emerge through a combination of complex overlapping mechanisms. Just as several processes in biology together distinguish living from non-living things, growing evidence from the contemporary scientific study of consciousness suggests that multiple mechanisms in neuroscience combine to separate conscious from non-conscious neural activity. Likewise, we can posit that consciousness is best understood through a synergistic combination of multiple neural mechanisms overlapping in space and time. Specifically, we hypothesize that systems crucial for consciousness include: (1) attention mechanism mediating signal detection, dynamic modulation of arousal, and bottom-up plus top-down attentional control, overlapping in space and time with; (2) systems that limit competing signals (e.g., through reduced default mode network activity); and, finally, (3) hierarchically organized systems that fully process signals for memory encoding and subsequent report^1^.

To investigate these multiple systems, a comprehensive approach is needed to identify activity throughout the brain at high spatial and temporal resolution specifically related to consciousness. We aimed to overcome several limitations of prior studies. For example, subcortical regions are poorly understood in human conscious perception and are often relegated to preconscious state-based precursors of consciousness^2–4^. We, therefore, investigated dynamic changes in both subcortical and cortical systems using techniques with complementary strengths. These included scalp electroencephalography (EEG) with large sample size and depth recordings from the human thalamus providing direct measurements of neural activity at high time resolution, and functional magnetic resonance imaging (fMRI) with large sample size analyzed with data-driven approaches providing comprehensive mapping of the whole brain. Linking these neural measures with consciousness, we used a threshold visual perception task to measure brain signals produced by physically identical stimuli that are either perceived versus not perceived, coupled with a unique innovation to remove the confound of overt report. When participants are asked to overtly report whether they have perceived a stimulus this introduces report-based post-perceptual processes (e.g., decision-making and motor planning) that can confound signals linked to consciousness, even when the report is delayed by several seconds after the stimulus^5–7^.

## Results

### Identifying visual conscious perception without report

**Fig. 1:**
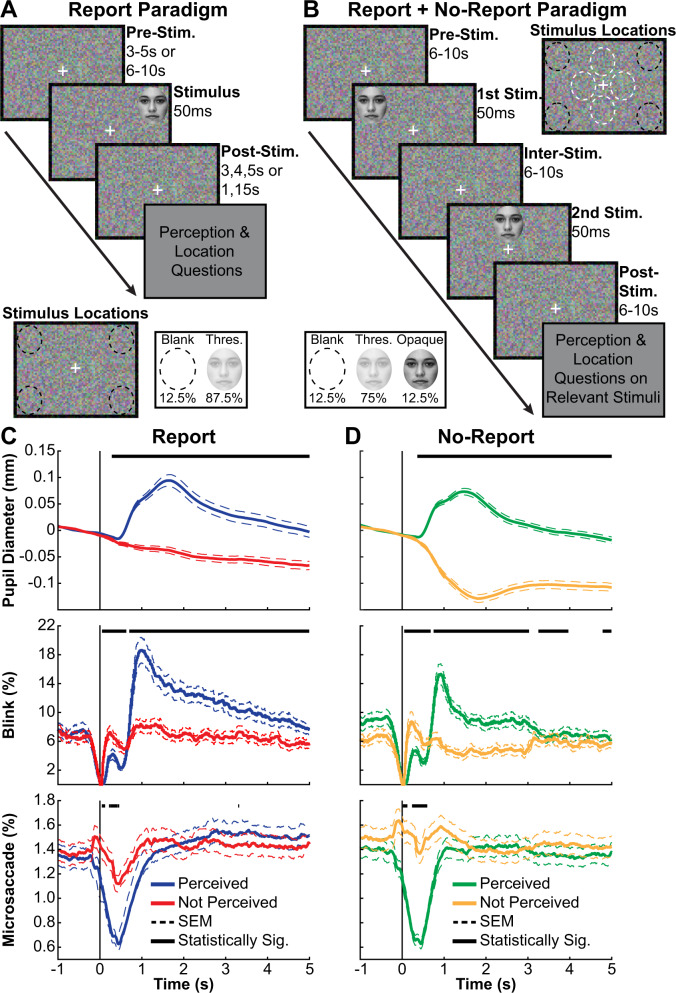
Report-dependent and report-independent behavioral paradigms for conscious perception; similar pupillometry and eye tracking responses. **A** Report Paradigm trial sequence, stimulus locations, and opacity conditions. **B** Report + No-Report Paradigm trial sequence, stimulus locations, and opacity conditions. **C** Report (task-relevant) data with perception based on overt participant responses and **D** no-report (task-irrelevant) data with perception classified by machine learning yield similar pupil, blink, and microsaccade dynamics (Full details on methods and materials are available in the Supplementary Information). Data in **C** and **D** are from stimuli at perceptual threshold (see **B** inset) in the Report + No-Report Paradigm (*N* = 68 participants). Stimulus onset was at time = 0 seconds (s). Classification score threshold for **D** was 0 (Fig. S9). Statistically significant (Statistically Sig.) different time points between perceived and not perceived data are indicated (black bars above pupil, blink, and microsaccade timecourses) based on cluster-based permutation tests (*p* < 0.05) (Full details on methods and materials are available in the Supplementary Information). Milliseconds (ms). Standard error of the mean (SEM). The face stimulus (**A**, **B**) is sourced from the FACES database (Ebner, N. C., Riediger, M., & Lindenberger, U. (2010). FACES—A database of facial expressions in young, middle-aged, and older women and men: Development and validation. Behavior Research Methods, 42, 351–362. doi:10.3758/BRM.42.1.351).

To address the confound of report-based post-perceptual processing we developed a novel no-report paradigm using transient changes in pupillometry and eye tracking to classify stimuli as perceived or not perceived without overt report (Figs. S1–9; Tables S4–6; full details on the methods and materials are available in the Supplementary Information). In a previously established Report Paradigm^8^, participants were repeatedly shown identical faces at 50% perceptual threshold (Fig. 1A). This resulted in approximately equal numbers of perceived and not perceived stimuli based on overt report of stimulus presence and location (Figs. S6, 7). We formed a novel combined Report + No-Report Paradigm by maintaining the report, task-relevant stimuli from the Report Paradigm and introducing identical no-report, task-irrelevant faces (i.e., stimuli that did not require overt report) interleaved with task-relevant stimuli, distinguished by different screen locations (four quadrant locations and four central locations; Fig. 1B). For example, in one session participants were told to report only on stimuli appearing in the screen quadrant locations and not in the screen central locations (Fig. S1A; see the Supplementary Information for counterbalancing and full details). The perception of no-report stimuli was determined by classification of pupillometry and eye tracking during the task. Specifically, we found pupil dilation and blink rate increases, along with microsaccade rate decreases for consciously perceived visual stimuli, present irrespective of task relevance (Fig. 1C, D). Pupillometry and eye tracking have been previously shown to correspond with perception and have been implemented as covert measures of consciousness^9–11^. However, the dynamics reported here are unique because they do not rely on task sequence (e.g., in some prior studies no-report stimuli were always first)^12,13^, changes in the stimulus (our stimuli are all identical), perceptual switching (e.g., binocular rivalry)^9^, nor stimulus type (e.g., stimulus modality), as we have found similar pupil, blink, and microsaccade responses for perceived auditory and tactile stimuli^14,15^.

### Report-independent event-related potentials in conscious visual perception

**Fig. 2:**
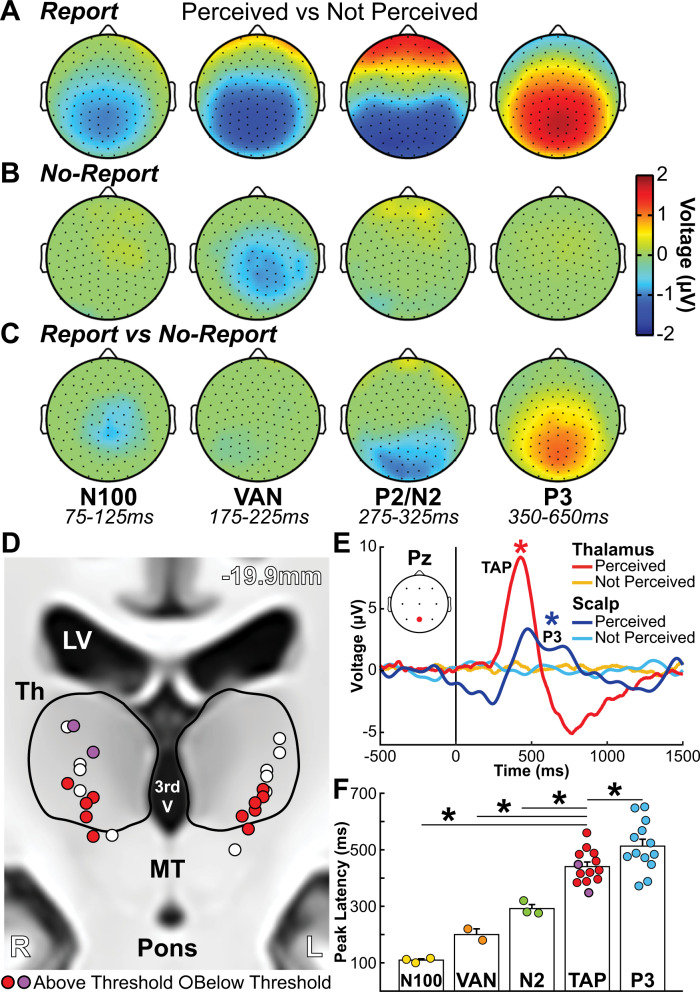
Cortical and thalamic electrophysiology signals in conscious perception. **A**–**C** Scalp topographical plots of high-density scalp EEG showing mean voltage (microvolts; µV) for all statistically significant samples by cluster-based permutation tests (*p* < 0.05) in four time windows corresponding with the event-related potentials (ERPs) N100 (75-125 milliseconds; ms), visual awareness negativity (VAN; 175–225 ms), P2/N2 (275–325 ms), and P3 (350–650 ms) for perceived versus not perceived stimuli in **A** report, **B** no-report, and **C** report versus no-report data. The VAN is present both for report and no-report data, whereas later ERPs, especially N2 and P3 are report-dependent. **D** Subcortical depth channel locations collapsed onto a coronal slice (−19.9 mm) in MNI brain template space. Red/purple (*N* = 13) and white bipolar channels (*N* = 11) distinguish above or below voltage threshold channels, respectively (Fig. S12) (Full details on methods and materials are available in the Supplementary Information). Red channels are with the RNS System (NeuroPace, Inc.) and purple channels with Natus NeuroWorks (Natus, Inc.) depth electrode recordings (Table S2). Neighboring anatomy includes lateral ventricles (LV), third ventricle (3rd V), thalamus (Th), midbrain tegmentum (MT), and pons. Right cortical hemisphere (R) and left cortical hemisphere (L). **E** Thalamic awareness potential (TAP) and P3 are seen, respectively in thalamic above-threshold contacts and Pz (location on inset) scalp EEG contacts. Mean timecourses show significant differences for perceived versus not perceived stimuli in the ERP analysis for TAP and P3 by cluster-based permutation tests (*p < 0.05) (Full details on methods and materials are available in the Supplementary Information). Stimulus onset was at time = 0 ms. **F** Peak latencies from stimulus onset for scalp ERPs and TAP. Circles represent individual data (mean channel latencies) and error bars indicate standard error of the mean (SEM). Significantly different latencies from TAP found by two-sided Wilcoxon rank sum test with Holm–Bonferroni correction (*p < 0.05; N100 versus TAP *p* = 0.014; VAN versus TAP *p* = 0.038; N2 versus TAP *p* = 0.014; P3 versus TAP *p* = 0.040; all *p* values are Holm-Bonferroni corrected). **A**–**C** Data for report stimuli are from Report Paradigm (*N* = 57) and Report + No-Report Paradigm (*N* = 65); data for no-report stimuli are from Report + No-Report Paradigm (*N* = 65). **D**–**F** Data are from Report Paradigm in patient participants with thalamic depth electrodes (*N* = 7).

Our first goal was to investigate report-dependent and report-independent brain signals at high time resolution. We found in a healthy, adult population (Report Paradigm: *N* = 57; Report + No-Report Paradigm: *N* = 65) the following well established event-related potentials (ERPs) for perceived stimuli in the report data, in temporal sequence: (1) N100, (2) VAN (visual awareness negativity), (3) P2/N2, and (4) P3/LP (late potentials) (Fig. 2A; Figs. S10A, 11A)^7,16–18^. The earliest ERP (N100) is thought to be related to visual cortical activation and was present for all stimuli regardless of perceived, not perceived, report or no-report, although the magnitude varied depending on conditions (Figs. S10, 11). The VAN was significantly greater for perceived versus not perceived stimuli, both for report and no-report data (Fig. 2A, B; Fig. S10A, C). Importantly, the VAN was not significantly different for report versus no-report data, whereas the later ERPs (P2/N2 and P3/LP) were significantly larger for perceived stimuli in the report data (Fig. 2C; Fig. S10E). Our novel paradigm thus strengthens previous findings that early ERPs, particularly the VAN, are seen even under no-report or task-irrelevant conditions^19^. Therefore, these early signals reflect the scalp neurophysiological signatures for report-independent conscious perception^20–22^. Furthermore, report and task-relevance introduce report-dependent changes dominated by later ERPs, including P3/LP that extend persistent neurophysiological activity for over 1 s after stimulus onset (Fig. S11A; see also ref. 23 and Fig. S9 in ref. 8). This replication of ERP findings from previous overt report and no-report studies validates and provides evidence that the current Report + No-Report Paradigm coupled with our novel covert measure of consciousness is at least comparable to other methods in the literature for limiting report-dependent signals.

### Thalamic awareness potential

Having established early report-independent signals of conscious perception and later report-dependent signals likely related to post-perceptual processing, we next sought to investigate subcortical signals selective for conscious perception and to determine their timing. A key subcortical brain structure for arousal and consciousness is the intralaminar thalamus^24,25^. More broadly, the thalamus is an essential regulator of cortical states, including arousal^26,27^. Likewise, anesthesia drugs commonly act on the thalamus to induce unconsciousness, while stimulating the thalamus under anesthesia can restore wakefulness^28,29^. This role of the thalamus is made possible by widespread and recurrent signals between the thalamus, particularly from the intralaminar thalamus, and cortex that modulate and coordinate cortical network activity^24,30,31^. In perception, the thalamus helps establish the sustained state-based precursors of consciousness and signal propagation, although the precise function of the thalamus in dynamic or transient modulation of conscious perception is unknown^32,33^. Building on this research, we were interested to directly study transient signals in the human thalamus in relation to the perception of sensory events.

We recruited, adult patient participants (Report Paradigm: *N* = 7) with chronically implanted deep brain recording and stimulation devices (RNS® System, NeuroPace, Inc.; Natus NeuroWorks, Inc.) for the treatment of epilepsy, providing unique access to this region (Full details on methods and materials are available in the Supplementary Information). We simultaneously recorded cortical electrophysiology from scalp EEG and subcortical signals from thalamic depth contacts (Fig. 2D; Figs. S12, 13; Tables S2, 3) while participants completed the Report Paradigm. We found a biphasic thalamic awareness potential (TAP) highly selective for perceived stimuli with an onset at ~250 ms and initial peak at ~430 ms post-stimulus presentation (Fig. 2E). TAP was localized to channels within or along the lateral border of the intralaminar thalamus (Fig. S2D; Fig. S12). TAP was also selectively present for perceived *auditory* stimuli in one participant (participant 1 in Table S2) who completed an analogous perceptual threshold auditory task in a separate study from our group (Fig. S14)^14^.

We next investigated the timing of TAP relative to scalp ERPs. First, we noted that the scalp ERPs recorded in the patient participants were similar to those of the healthy participants, despite different recording systems and sample sizes (Fig. S13). We found that TAP preceded P3, but followed the N100, VAN, and N2 (Fig. 2F). Therefore, the timing of TAP was later than ERPs found in the prior experiment to be report-independent (VAN) and fell within or earlier than ERPs that were report-dependent (N2, P3). We did not directly test whether TAP was report-dependent because of limited recording time with the patient participants. However, because we hypothesized that TAP is one node in a broad subcortical arousal and salience network participating in attention state dynamics and consciousness^34,35^, we next used fMRI in a large cohort of healthy participants to investigate cortical and subcortical conscious perception-linked dynamics, with and without overt report.

### Report-independent fMRI changes in conscious visual perception

**Fig. 3:**
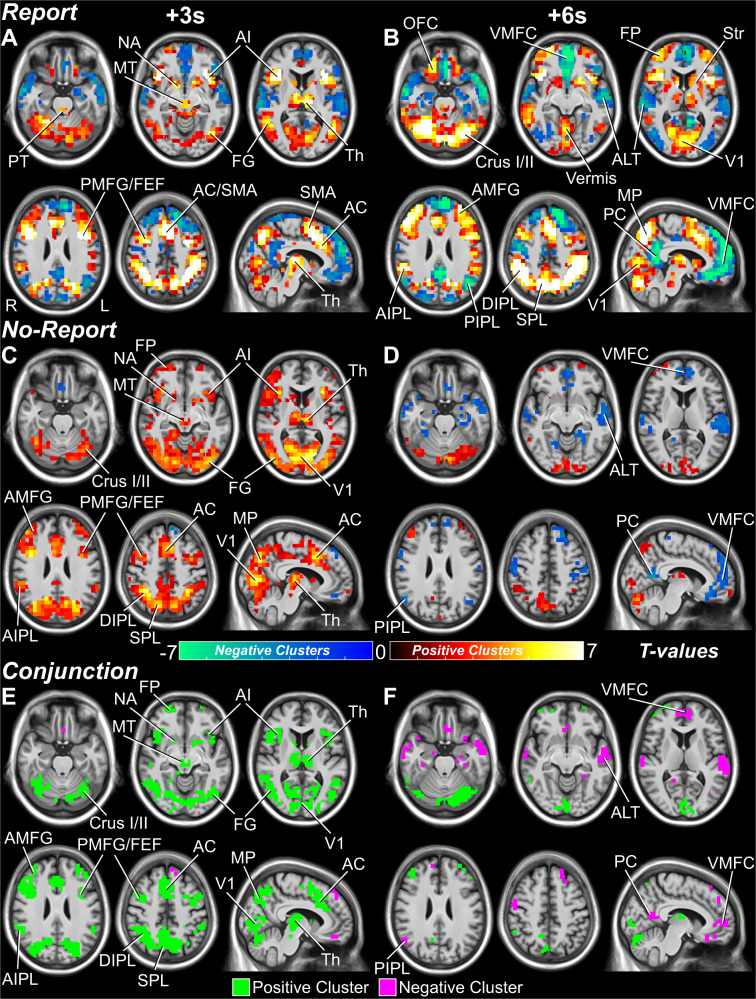
Whole brain fMRI maps for conscious perception with and without overt report. **A**, **B** Report and **C**, **D** no-report perceived minus not perceived statistically significant voxels from cluster-based permutation tests (*p* < 0.05) at 3 and 6 seconds (s) post-stimulus presentation (Full details on methods and materials are available in the Supplementary Information). Statistically significant positive and negative voxel cluster *t*-values are shown in warm and cool colors, respectively. Anatomical regions are labeled at their peak response between the 3 and 6-s time points. **E**, **F** Voxel-level conjunction analysis of report and no-report, perceived minus not perceived statistical whole brain maps with shared (report-independent) increases shown in green and shared decreases shown in purple. Pontine tegmentum (PT), midbrain tegmentum (MT), thalamus (Th), nucleus accumbens (NA), striatum (Str), lateral superior cerebellum (Crus I/II), anterior insula/claustrum (AI), anterior cingulate (AC), supplementary motor area (SMA), primary visual cortex (V1), fusiform gyrus (FG), anterior middle frontal gyrus (AMFG), posterior middle frontal gyrus (PMFG), frontal eye fields (FEF), frontal pole (FP), orbital frontal cortex (OFC), ventral medial prefrontal cortex (VMFC), anterior inferior parietal lobule (AIPL), dorsal inferior parietal lobule (DIPL), posterior inferior parietal lobule (PIPL), superior parietal lobule (SPL), medial parietal cortex (MP), posterior cingulate/precuneus (PC), and anterolateral temporal cortex (ALT). Right cortical hemisphere (R) and left cortical hemisphere (L). See Slides S1, 2, and 4^39^ for all times 20 s pre and post-stimulus presentation for report, no-report, and conjunction results. Data for report stimuli are from the Report Paradigm (*N* = 34) and Report + No-Report Paradigm (*N* = 65); data for no-report stimuli are from the Report + No-Report Paradigm (*N* = 65). Results are visualized on the brain volume PD25-MPRAGET1w-template-500um.nii (0.5 mm^3^ voxels) from an MNI registration with the BigBrain dataset (https://osf.io/xkqb3/)^61,62^.

**Fig. 4:**
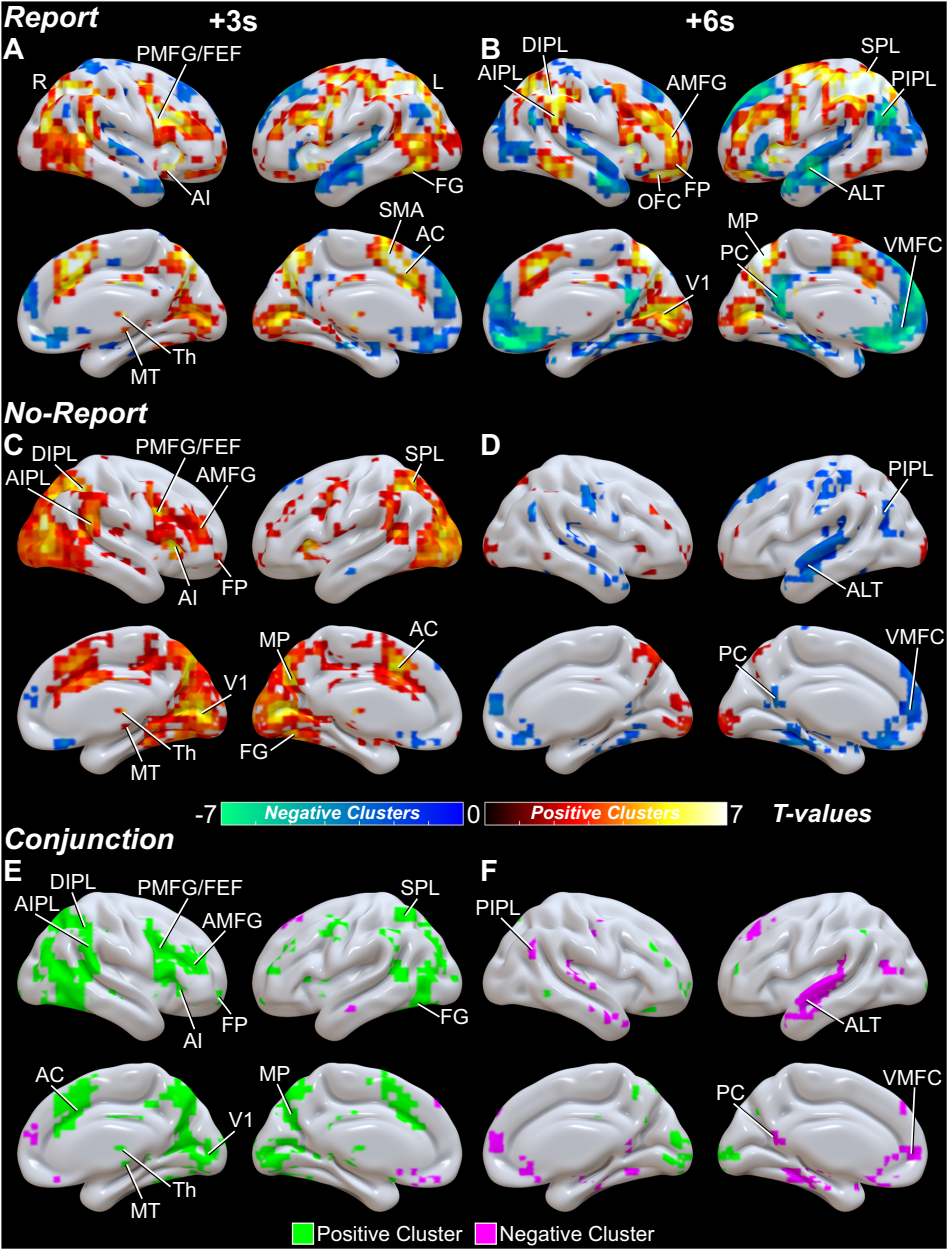
Whole brain fMRI surface maps for conscious perception with and without overt report. The same results presented in Fig. 3 plotted on an inflated MNI ICBM152 anatomical brain surface mesh. **A**, **B** Report and **C**, **D** no-report perceived minus not perceived statistically significant voxels from cluster-based permutation tests (*p* < 0.05) at 3 and 6 seconds (s) post-stimulus presentation (Full details on methods and materials are available in the Supplementary Information). Statistically significant positive and negative voxel cluster *t*-values are shown in warm and cool colors, respectively. Anatomical regions are labeled at their peak response between the 3 and 6-s time points. **E**, **F** Voxel-level conjunction analysis of report and no-report, perceived minus not perceived statistical whole brain maps with shared (report-independent) increases shown in green and shared decreases shown in purple. Midbrain tegmentum (MT), thalamus (Th), anterior insula/claustrum (AI), anterior cingulate (AC), supplementary motor area (SMA), primary visual cortex (V1), fusiform gyrus (FG), anterior middle frontal gyrus (AMFG), posterior middle frontal gyrus (PMFG), frontal eye fields (FEF), frontal pole (FP), orbital frontal cortex (OFC), ventral medial prefrontal cortex (VMFC), anterior inferior parietal lobule (AIPL), dorsal inferior parietal lobule (DIPL), posterior inferior parietal lobule (PIPL), superior parietal lobule (SPL), medial parietal cortex (MP), posterior cingulate/precuneus (PC), and anterolateral temporal cortex (ALT). See Fig. 3 for additional changes in subcortical structures not visible in surface renderings (midbrain tegmentum, thalamus, pontine tegmentum, striatum, nucleus accumbens, cerebellar Crus I/II, cerebellar vermis). Right cortical hemisphere (R) and left cortical hemisphere (L). Data for report stimuli are from the Report Paradigm (*N* = 34) and Report + No-Report Paradigm (*N* = 65); data for no-report stimuli are from the Report + No-Report Paradigm (*N* = 65). Results are visualized on the brain surface mesh image BrainMesh_ICBM152_smoothed from Surf Ice (Version 12.1; https://www.nitrc.org/projects/surfice/).

Previous studies investigating conscious perception with fMRI have focused on cortical dynamics, with fewer reporting on subcortical signals^9,34,36–38^. No-report or task-irrelevant fMRI studies have contributed to the debate on the role of association cortices in perception versus post-perceptual processes^9,12,38^. Meanwhile, there is little insight on the dynamic of subcortical networks in a no-report or task-irrelevant condition in conscious perception. Extending from these previous studies, we implemented our report-dependent and report-independent behavioral paradigms with fMRI in a healthy, adult population (Report Paradigm: *N* = 34; Report + No-Report Paradigm: *N* = 65). This revealed a broad network of subcortical and cortical regions showing report-independent fMRI changes especially at earlier times after stimulus presentation. Perceived versus not perceived blood-oxygen-level-dependent (BOLD) responses were distinguished by early (<4 s) and late (>5 s) dynamics analyzed by spatiotemporal cluster-based permutation tests (Full details on methods and materials are available in the Supplementary Information). At 3 s post-stimulus (Fig. 3A, C and Fig. 4A, C; Fig. S15A, C; Slides S1, 2^39^), report-independent increases were shared by both report and no-report data in signal detection regions, including primary visual cortex (V1), fusiform gyrus (FG), and the posterior middle frontal gyrus (PMFG; near to the frontal eye fields, FEF). Additional report-independent subcortical and cortical increases were found at early times in arousal and salience networks, including the midbrain tegmentum (MT), thalamus (Th), nucleus accumbens (NA), anterior cingulate/supplementary motor area (AC/SMA), and anterior insula/claustrum (AI). Finally, shared report-independent increases at early times were seen in attention and executive control networks, including the anterior inferior parietal lobule (AIPL), dorsal inferior parietal lobule (DIPL), superior parietal lobule (SPL), medial parietal cortex (MP), anterior middle frontal gyrus (AMFG), frontal pole (FP), and cerebellum (Crus I, II).

In contrast, at 6 s post-stimulus and later there were fewer shared report-independent regions seen in both report and no-report data (Figs. 3B, D, 4B, D; Fig. S15B, D; Slide S1, 2^39^). Most shared changes at 6 seconds involved decreases in default mode network (DMN) areas, including the ventral medial prefrontal cortex (VMFC), posterior cingulate/precuneus (PC), posterior inferior parietal lobule (PIPL), and anterolateral temporal cortex (ALT). Shared increases at 6 s were limited to few and relatively small regions of V1, cerebellum, fronto-parietal cortex, and Th. At the same time, the report data alone showed prominent persistent or peak increases at 6 seconds in attention and executive control networks, including AIPL, SPL, MP, AMFG, orbital frontal cortex (OFC), FP, striatum (Str), and cerebellum, as well as, more prominent decreases in DMN regions (Figs. 3B, 4B; Fig. S15B; Slide S1^39^).

Conjunction analyses emphasize the broad shared report-independent networks seen mainly at earlier times in fMRI, including regions important for signal detection, arousal, salience, attention, and executive control, as well as, some involvement of DMN (Figs. 3E, F, Fig. 4E, F; Slide S4^39^). Report versus no-report statistical comparisons with cluster-based permutation tests (*p* < 0.05) of perceived minus not perceived fMRI signal show greater differences at later times, including in left cortical motor regions, possibly linked to motor planning for subsequent right-hand behavioral responses (Fig. S16A, B; Slide S3^39^). Likewise, analysis of signals significant only in report (Fig. S16C, D; Slide S5^39^) highlighted the separate report-dependent regions such as motor cortex, OFC, Str, and PC, not significantly involved at any time in no-report data, as well as, attention, executive, and cerebellar regions that were shared at early times, but remained persistently activated only in the report data. Analysis of signals significant only in no-report data found early increases in sensory regions (e.g., V1 and FG) that are unique to the no-report network (Fig. S16E, F; Slide S6^39^).

### Eye movement-based event-related potentials and fMRI changes

In the results presented above, we used various eye measurements, including pupil size, spontaneous blinks, and microsaccades to predict conscious visual perception without overt report. However, in addition to signals specific for conscious visual perception, eye movement-related signals may also correspond with neural activity reflecting the following: (1) transient changes in general conscious perception state not specific to visual stimuli and (2) changes related to eye movement motor control^40–44^. To separately identify the different types of eye movement-related signals, we analyzed the hdEEG and fMRI changes without visual task stimulus presentation in two different ways. First, we used eye movements to identify transient events which may reflect spontaneous increases in perceptual conscious awareness but without a visual stimulus. Second, to identify separate motor signals for pupil, blink, or microsaccade control without a visual stimulus, we looked at each of these three signals separately.

In the first analysis, aimed at transient conscious awareness without visual stimuli, the interstimulus intervals (ISI) after each stimulus presentation in the Report + No-Report Paradigm were classified as “perceived” or “not perceived” using the same classification approach implemented on the no-report data (Full details on methods and materials are available in the Supplementary Information). While there were no task stimuli during the ISI, the ISI classified “perceived” and “not perceived” trials had similar pupil, blink, and microsaccade timecourses as those for report and no-report data (Fig. S17A).

**Fig. 5:**
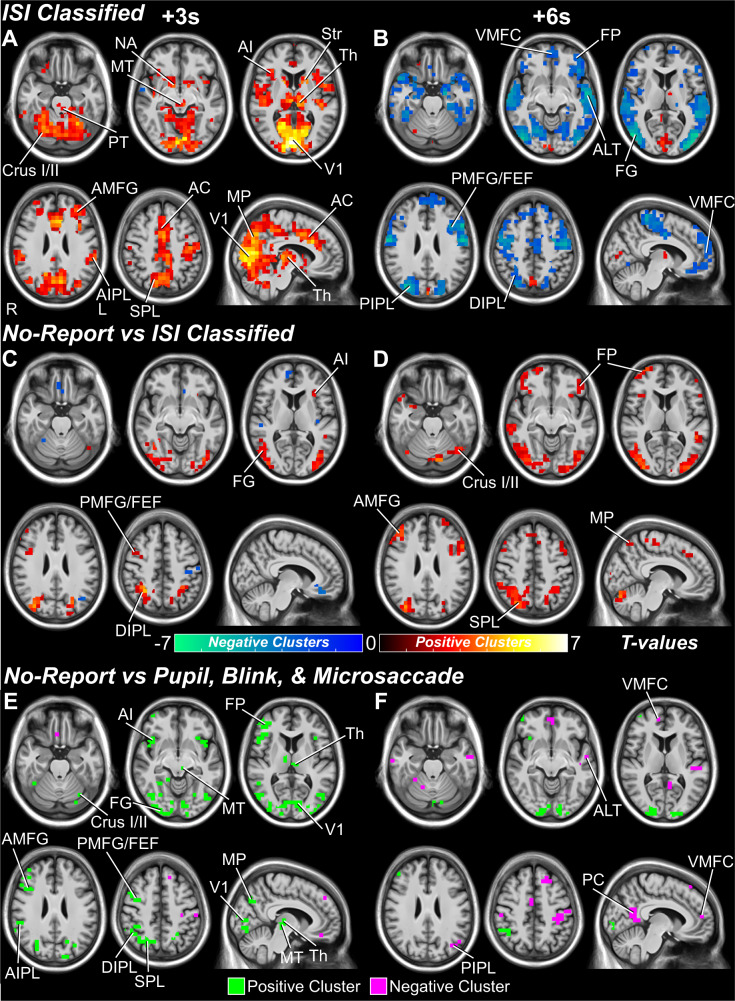
Whole brain fMRI maps for interstimulus interval (ISI) classified data and for eye-movement comparisons with no-report data. **A**, **B** ISI classified “perceived” minus “not perceived” and **C**, **D** no-report perceived minus not perceived versus ISI classified “perceived” minus “not perceived” data. Statistically significant voxels are shown from cluster-based permutation tests (*p* < 0.05) at 3 and 6 seconds (s) post-stimulus presentation for no-report data or post the ISI center time (Full details on methods and materials are available in the Supplementary Information). Statistically significant positive and negative voxel cluster *t*-values are shown in warm and cool colors, respectively. Anatomical regions are labeled at their peak response between the 3 and 6-second time points. **E**, **F** No-report perceived minus not perceived data with removal of pupil, blink, and microsaccade-related fMRI signals by voxel-level conjunction analysis (Full details on methods and materials are available in the Supplementary Information). Whole brain maps with increases shown in green and decreases shown in purple. Compare these results with Fig. 3E, F. Pontine tegmentum (PT), midbrain tegmentum (MT), thalamus (Th), nucleus accumbens (NA), striatum (Str), lateral superior cerebellum (Crus I/II), anterior insula/claustrum (AI), anterior cingulate (AC), primary visual cortex (V1), fusiform gyrus (FG), anterior middle frontal gyrus (AMFG), posterior middle frontal gyrus (PMFG), frontal eye fields (FEF), frontal pole (FP), ventral medial prefrontal cortex (VMFC), anterior inferior parietal lobule (AIPL), dorsal inferior parietal lobule (DIPL), posterior inferior parietal lobule (PIPL), superior parietal lobule (SPL), medial parietal cortex (MP), posterior cingulate/precuneus (PC), and anterolateral temporal cortex (ALT). Right cortical hemisphere (R) and left cortical hemisphere (L). Data are from the Report + No-Report Paradigm (*N* = 65). Results are visualized on the brain volume PD25-MPRAGET1w-template-500 μm.nii (0.5 mm^3^ voxels) from an MNI registration with the BigBrain dataset (https://osf.io/xkqb3/)^61,62^.

Next, we implemented the same hdEEG and fMRI report and no-report data analyses on the classified ISI data. We found no ERPs associated with the classified “perceived” and “not perceived” ISI data (Fig. S18). The lack of ERPs could be due to insufficient temporal precision in eye movement-based event onset and the relative insensitivity of ERPs to subcortical signals; both limitations could be overcome with fMRI. Indeed, fMRI changes for ISI classified “perceived” minus “not perceived” data revealed statistically significant increases and decreases in many of the same regions as the no-report data despite the absence of a visual stimulus (Fig. 5A, B). At 3 s post the ISI trial center time, increases in arousal and salience networks were seen, including PT, MT, NA, Str, AI, AC, and Th (Fig. 5A). These findings concur with previous literature that shows arousal and salience network activity associated with spontaneous changes in pupil size and blink rate (e.g., refs. 41,43). In addition, increases were seen at 3 s in attention and executive control networks, including AMFG, AIPL, SPL, and cerebellum. At 6 s, there were statistically significant decreases in PMFG/FEF, FP, SMA, and DMN regions, including VMFC, DIPL, and ALT (Fig. 5B). Notably, the overlap in fMRI changes between no-report and ISI was not total. No-report data (with visual stimuli) showed significantly greater increases than ISI classified data (without visual stimuli) in visual detection and attention regions including FG, DIPL, and PMFG/FEF at 3 s (Fig. 5C) and in FP, AMFG, MP, SPL, and Crus I/II at 6 s (Fig. 5D).

In the second analysis, we aimed to identify the fMRI changes for pupil, blink, and microsaccade dynamics independently without visual stimuli (Full details on methods and materials are available in the Supplementary Information). Notably, when ISI events were sorted based only on one type of eye movement (e.g., for pupils only see Fig. S17B), minimal changes were seen in the other two eye movement types, suggesting relative independence of pupil, blink, and microsaccade-related signals in the respective sorted data sets (see also Fig. S17C, D). In agreement with this relative independence of signals, the no stimulus (ISI) pupil-sorted data showed increases mainly in arousal, salience, and primary visual networks (Fig. S19A, B); blink-sorted data showed increases mainly in primary visual cortex and a few other regions, including AC and AI (Fig. S19C, D); and microsaccade-sorted data showed relatively few changes in small regions of FP, AMFG, SPL, and Crus I/II (Fig. S19E, F).

To remove these three independent eye movement control-related signals from the no-report data, we performed a contrast analysis of no-report versus pupil, blink, and microsaccade-sorted data, followed by a conjunction of these three separate analyses (Full details on methods and materials are available in the Supplementary Information). This revealed that the report-independent visual conscious network without eye movement signal contributions maintains a large cortical and subcortical network of visual consciousness-linked activity (Fig. 5E, F). The major networks observed in no-report data were largely preserved, including increases at 3 seconds in detection, arousal, and salience regions, including MT, Th, AI, PMFG/FEF, FG, and V1, as well as in attention and executive control regions, including AMFG, DIPL, AIPL, SPL, FP, and Crus I/II (Fig. 5E). At 6 s, later decreases were found in the DMN (Fig. 5F). Overall, although somewhat less in extent, the regions found in the no-report data with eye movement signals removed were similar to the conjunction analysis of report and no-report data (compare Fig. 3E, F and Fig. 5E, F).

### Major cortical and subcortical networks in conscious visual perception

**Fig. 6:**
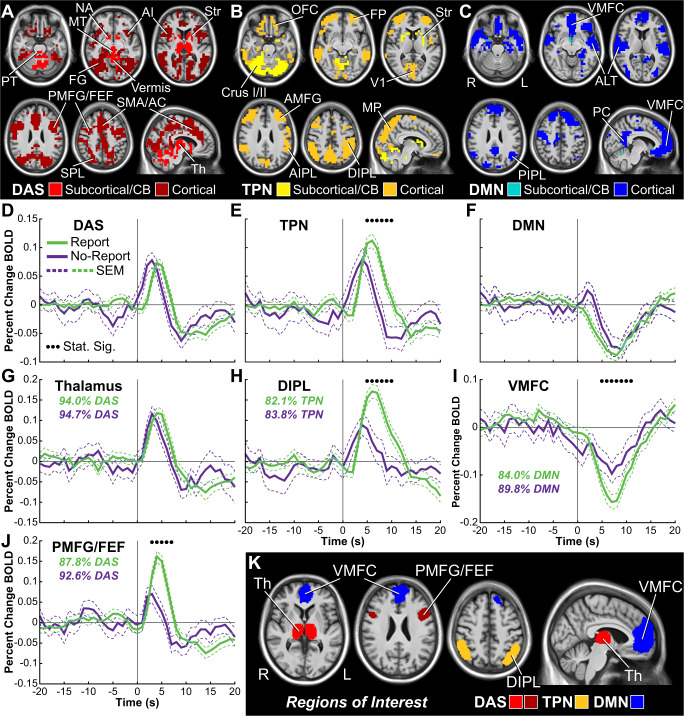
Data-driven anatomical clustering of fMRI signals for conscious perception and region of interest (ROI) timecourses. **A**–**C** Three large-scale networks found with k-means clustering based on fMRI timecourses (Fig. S20). **A** Detection, arousal, and salience networks (DAS), **B** task-positive networks (TPN), and **C** default mode network (DMN). Cortical and subcortical/cerebellar territories are shown in different shades. **D**–**F** Mean percent change blood-oxygen-level-dependent (BOLD) timecourses for report (green) and no-report (purple) perceived minus not perceived conditions for DAS, TPN, and DMN. **G**–**J** Selected subregion mean percent change BOLD timecourses (see also Figs. S21–23). Percentages of voxels from a particular network (DAS, TPN, or DMN) versus all network voxels found in each ROI are shown for report (green) and no-report (purple) data (Full details on methods and materials are available in the Supplementary Information). **K** Anatomical visualization of subregion ROIs from **G**, **H**, **I**, and **J**. Pontine tegmentum (PT), midbrain tegmentum (MT), thalamus (Th), nucleus accumbens (NA), striatum (Str), anterior insula/claustrum (AI), anterior cingulate (AC), supplementary motor area (SMA), primary visual cortex (V1), fusiform gyrus (FG), anterior middle frontal gyrus (AMFG), posterior middle frontal gyrus (PMFG), frontal eye fields (FEF), frontal pole (FP), orbital frontal cortex (OFC), ventral medial prefrontal cortex (VMFC), anterior inferior parietal lobule (AIPL), dorsal inferior parietal lobule (DIPL), posterior inferior parietal lobule (PIPL), superior parietal lobule (SPL), medial parietal cortex (MP), posterior cingulate/precuneus (PC), and anterolateral temporal cortex (ALT). Right cortical hemisphere (R) and left cortical hemisphere (L). Stimulus onset was at time = 0 seconds (s). Data for report stimuli are from the Report Paradigm (*N* = 34) and Report + No-Report Paradigm (*N* = 65); data for no-report stimuli are from the Report + No-Report Paradigm (*N* = 65). Clusters (A-C) and ROIs (K) are visualized on the brain volume PD25-MPRAGET1w-template-500um.nii (0.5 mm^3^ voxels) from an MNI registration with the BigBrain dataset (https://osf.io/xkqb3/)^61,62^.

To further investigate the main large-scale networks involved in conscious perception with and without report and the temporal profile of these networks, we used temporal correlation-based k-means clustering across the entire brain (Full details on methods and materials are available in the Supplementary Information). Data-driven clustering of statistically significant voxels for report perceived versus not perceived fMRI signals revealed three anatomically and functionally distinct networks: (1) early positive, (2) late positive, and (3) late negative (Fig. 6A–F; Fig. S20D, E, F). The early positive network has a peak at 3–4 s after stimulus onset and includes subcortical and cortical detection, arousal, and salience networks (DAS; Fig. 6A, report trace in Fig. 6D). They include FG, PMFG/FEF, MT, Th, NA, AC/SMA, AI, cerebellar vermis, and subregions of the Str, AIPL, SPL, and MP (Fig. 6A). The late positive network peaks ~6 s after stimulus onset and includes task-positive networks (TPN) such as AMFG, OFC, FP, cerebellum Crus I and II, and subregions of the Str, AIPL, SPL, and MP (Fig. 6B; report trace in Fig. 6E). The late negative network has a trough at 6–8 s after stimulus onset and occupies DMN regions that are exclusively cortical, including the VMFC, PC, PIPL, and ALT (Fig. 6C; report trace in Fig. 6F). Thus, three major and distinct brain networks for conscious perception emerge from the fMRI data entirely based on BOLD timecourse dynamics.

How does overt report change the signal timecourses in these networks? Analysis of mean timecourses for all voxels within each network revealed that only TPN was on the whole different between report and no-report data at later times (Fig. 6D–F). However, subregion analyses showed that all three networks contained both regions that agreed and regions that differed between report and no-report data (Fig. 6G–J; Figs. S21–23). Importantly, the fMRI timecourse for Th (>94% DAS voxels) did not differ between report and no-report conditions (Fig. 6G). In contrast, the PMFG/FEF (>87% DAS voxels), DIPL (>82% TPN voxels), and VMFC (>79% DMN voxels) had greater signal amplitude and duration for report versus no-report conditions (Fig. 6H–J). Similar to the conjunction and difference analyses already discussed (Figs. 3E, F, 4E, F; Fig. S16), additional timecourse analyses confirmed that some brain areas (e.g., left motor cortex; Fig. S22) are only significantly involved in report data, whereas many more regions are shared between report and no-report data at early times, but show persistent or larger signals in report data at later times (e.g., DIPL, SPL, MP; Figs. S21–23).

## Discussion

We found that multiple cortical and subcortical systems are involved during conscious perception of visual stimuli independent of report. Report-independent signals for visual conscious perception are early and transient. In EEG, they include the N100 and VAN. Report-independent signals from fMRI, with the identical behavioral paradigm and many of the same participants as EEG, include early and transient changes in widespread regions of three major subcortical and cortical brain networks. The importance of subcortical networks in conscious perception was further supported by our identification of the TAP through direct recordings from the human thalamus. Meanwhile, report-dependent signals are late and persistent, and on EEG include the P2/N2 and P3/LP; and late (~6 s) fMRI signals in DAS, TPN, and DMN. Persistent BOLD signals for report data may be linked to prolonged neuronal activity changes needed for subsequent report, manifested electrophysiologically as late potentials (Fig. S11A), or other sustained activity following perception^8,23^.

These findings support an approach to studying consciousness with a broad scope—investigating multiple overlapping systems in neuroscience to capture the full complexity of neurophysiology that we and others measure experimentally during conscious perception. Our identification of specific report-independent subcortical and cortical systems overlapping in space and time for conscious perception can be placed along a proposed timeline consistent with the three major networks found in our data^1^. First, (1) *Detection/arousal/salience (DAS)*: when a visual stimulus appears, activation of V1 interacts with FG, FEF, and other regions for signal detection^45–48^. A dynamic transient pulse in subcortical arousal (e.g., MT and Th) and emotional/motivational systems (e.g., NA) amplifies and facilitates bottom-up attentional salience and top-down attentional control^35,49,50^. Next, (2) *Default-mode network (DMN)*: switching off of the DMN and related circuits can reduce competing signals to prevent interference with conscious perception^8,51–53^. Finally, (3) *Task-positive networks (TPN)*: a broad wave of hierarchical processing sweeps through task-positive cortical and subcortical areas to fully process the event before it is encoded in working and episodic memory systems. The later events along this timeline, especially for TPN, merge into report-dependent, post-perceptual processing needed for subsequent report. Overall these current results agree with recent work suggesting that visual perception begins with early signal detection^46^ followed by a pulse of arousal, attentional, and limbic signal amplification^35^ leading to network switching and a wave of processing to form conscious experiences^1,8^.

Our data-driven DAS network overlaps with the salience network found in brain imaging studies during resting state and numerous tasks^50^. While previous discussion on the salience network emphasizes its role in cognition, emotive, and interoceptive function, these results also support its role in sensory perception. While there have long be theories of consciousness that include subcortical networks, the cortex is commonly considered dominant in studies of consciousness and cognition^54^. Our results encourage considering cortical and subcortical networks as proportional actors in sensory perception. This is further supported by lesion cases in the brainstem, insula, and thalamus that suggest a causal role of these arousal and salience network sites in sensory consciousness^25,55–57^. Still, cortical lesions that result in sensory perception impairment such as visual agnosia maintain an essential role for the cortex in consciousness^58^, and this is further supported by a widespread report-independent cortical signal in the current study.

For reasons of statistical power and correspondence with previous literature, in the current investigation, we used a single visual face stimulus across multiple brain measurement techniques and report/no-report conditions with large sample size. The experiment we performed does not allow us to differentiate between brain activity related to conscious visual perception of faces or of the particular face we used versus more general activity for conscious visual or other sensory perception. The networks reported in this study likely represent a combination of unimodal and multimodal systems. Therefore, in addition to well-known visual regions (e.g., V1 and FG), the reported networks could be interpreted as representing the underlying neural mechanisms of sensory consciousness more broadly, not only vision. For example, we found the VAN as a report-independent response and which has also been replicated in auditory and somatosensory perception, indicating a general perceptual awareness negativity^19^. This interpretation is especially relevant to the report-independent association or multimodal systems we observed, including arousal, attention, detection, DMN, and salience networks. Recent icEEG work from our group and prior fMRI work by others have found overlapping network responses with different sensory modalities such as auditory perception to those reported here in visual conscious perception^14,38^. In addition, we reported one patient participant here who had similar thalamic awareness potential responses with visual and auditory perception (Fig. S14). Future work will be needed with report and no-report paradigms using different stimuli and sensory modalities to identify networks for particular stimuli versus networks more generally purposed for sensory perception.

The eye movement-based findings share at least two important messages for future consideration. First, because healthy, alert participants are conscious throughout an experiment, not just during a perceived target stimulus, the classification of ISI trials as perceived may indicate genuine instances of perception, although not of the task stimuli nor necessarily of visual sensory content. Therefore, the ISI classified data results, along with previous findings on eye movement-based neural activity may reveal a portion of the consciousness network that is shared among the senses. This interpretation is bolstered in the current findings that show that visual, content specific, and detection regions present in the no-report data, are absent in the ISI classified data (e.g., FG, PMFG/FEF; Fig. 5C, D). The second lesson is derived from sorting of ISI data based on pupil, blink, and microsaccades separately to obtain fMRI signals related to each of these eye movements. These results demonstrate that experimental tasks may systematically introduce eye movement changes which could influence fMRI findings (Fig. S19). Importantly, eye movement-based dynamics do not preclude those regions as members of the consciousness network. Still, the interpretation of previous results and future studies should be wary of this potential confound. In the current study, we find that without report and even after removing eye movement-based confounds, the visual consciousness network remains broadly distributed in subcortical and cortical networks.

A limitation of the current investigation is that while the Report + No-Report Paradigm eliminates the demands of overt report, participants were free to covertly engage in report-dependent processes^59^. Moreover, there are other non-report-based sources of post-perceptual processing (e.g., self-monitoring and executive functions) that may also contaminate the targeted dynamics specific to consciousness^5,6^. Together, these considerations suggest that in the no-report condition there can still be post-perceptual processes confounding the perceptual signals of interest. These challenges are partially muted because the Report + No-Report Paradigm occupied participants with a distractor task concurrent to the no-report condition, thereby reducing the opportunity for an engaged participant to covertly apply cognitive processes on the no-report stimuli. Nevertheless, these challenges are broadly relevant to no-report studies and future research should endeavor to consider strategies to manage these potential sources of confound to uncover the targeted perception-linked signals with even greater specificity.

Brain mechanisms of conscious visual perception are early, transient, and large-scale. The spatiotemporal signatures of conscious perception suggest that the human brain produces consciousness using synergistic and redundant systems, combining signal detection, attentional amplification, selective information control, and processing. A large and layered neural architecture would be advantageous by making the conscious network resistant to damage or change. We found that even without report-based post-perceptual processing the neural mechanisms of conscious perception are widely distributed across cortical and subcortical sites. Investigating these rich and complex overlapping systems may provide a satisfactory explanation for consciousness.

## Methods

For full details of all methods and materials please see the Supplementary Information.

### Participants

The current investigation comprises two main participant groups, healthy and patient volunteers, from which six primary data sets were gathered: (1) healthy, Report Paradigm with fMRI, (2) healthy, Report Paradigm with hdEEG, eye tracking, and pupillometry, (3) patient, Report Paradigm with ldEEG and icEEG, (4) patient, Report Paradigm with icEEG, (5) healthy, Report + No-Report Paradigm with fMRI, eye tracking, and pupillometry, and (6) healthy, Report + No-Report Paradigm with hdEEG, eye tracking, and pupillometry (Table S1).

All study procedures were carried out in accordance to the Declaration of Helsinki and all participants gave informed consent for participating in the study procedures approved by the Yale University and University of Pittsburgh Institutional Review Boards. For all data gathered during the COVID-19 pandemic, adapted study procedures for reducing the risk of COVID-19 transmission were approved by Yale Environmental Health and Safety, including enhanced disinfection, social distancing, and personal protective equipment protocols.

#### Healthy participants

A total of 144 healthy, adult participants were recruited from Yale University and the New Haven, Connecticut communities. There were four primary data sets gathered from the healthy participants: (1) Report Paradigm with simultaneous fMRI recording (*N* = 37; mean age = 27.22 years; age range = 18–42 years; females = 17; right-handed = 35), (2) Report Paradigm with simultaneous hdEEG and binocular eye tracking and pupillometry recordings (*N* = 59; mean age = 26.20 years; age range = 19–43 years; females = 37; right-handed = 53), (3) Report + No-Report Paradigm with simultaneous fMRI and monocular eye tracking and pupillometry recordings (*N* = 65; mean age = 24.77 years; age range = 18–46 years; females = 39; right-handed = 64), and (4) Report + No-Report Paradigm with simultaneous hdEEG and binocular eye tracking and pupillometry recordings (*N* = 65; mean age = 24.58 years; age range = 18–46 years; females = 39; right-handed = 63). Summary information for the healthy participant data sets is reported in Table S1.

#### Patient participants

Seven adult participants with implanted thalamic depth electrodes for seizure monitoring and treatment were recruited from the University of Pittsburgh Medical Center Department of Neurology Epilepsy Division and the Yale-New Haven Hospital Comprehensive Epilepsy Program. Two primary data sets were gathered from the patient participants: (1) Report Paradigm with simultaneous low-density scalp EEG (ldEEG) and thalamic intracranial EEG (icEEG) (*N* = 6; mean age = 24.17 years; age range = 20-31; females = 4; right-handed = 6) and (2) Report Paradigm with icEEG alone, without concurrent ldEEG (*N* = 1; age = 29; female = 1; left-handed = 1). Summary information for the patient participant data sets is reported in Table S1.

For information about data exclusion please see the Supplementary Information *Behavioral Exclusions* and *Motion-Based Rejections* sections.

### Visual perception paradigms

Our goal was to develop a valid approach to investigate brain signals during conscious perception with versus without overt report. Briefly, our overall strategy was as follows: first, we developed a Report Paradigm^8^; next we developed a Report + No-Report Paradigm and confirmed the report data from both paradigms were similar; finally, we developed a machine learning approach to classify the no-report data in the Report + No-Report Paradigm as perceived or not perceived.

The two visual, perceptual threshold paradigms (Report and Report + No-Report Paradigms) were designed for the analysis of three primary perceptual contrasts: (1) compare report perceived versus not perceived visual stimuli (tested with the Report Paradigm and the report portion of the Report + No-Report Paradigm); (2) compare no-report perceived versus not perceived visual stimuli (tested with the no-report portion of the Report + No-Report Paradigm); and (3) compare (1) versus (2), or results from report versus no-report perceptual tasks.

#### Report paradigm

The Report Paradigm was previously administered and published by our group^8^. In summary, the paradigm consists of two sequential phases: (1) a perceptual threshold calibration and (2) testing phase. In both task phases, the target stimulus was a greyscale, neutral expression, human face (3.7 × 4.6 degrees) selected from the FACE database (Ebner, N. C., Riediger, M., & Lindenberger, U. (2010). FACES—A database of facial expressions in young, middle-aged, and older women and men: Development and validation. Behavior Research Methods, 42, 351–362. doi:10.3758/BRM.42.1.351). The target stimulus appeared for 50 ms in one of four pre-selected quadrant locations of the display screen (Fig. 1A). The background of the screen was filled with either a visual static noise or a nature documentary (BBC series Blue Planet episode “Coral Seas”). The documentary background was played with audio, aiming to mimic a naturalistic viewing environment. All participants experienced both background conditions in alternating task runs. The initial background used in a run (static noise or documentary) was counterbalanced across participants. In the documentary background condition, four rectangular static noise patches were shown in each of the quadrant locations of the screen where the target stimulus could appear to control the background image over which the target stimulus was presented. At the center of the screen was a white fixation cross (0.3 × 0.3 degrees). See the Supplementary Information for full details on the Report Paradigm.

#### Report + No-Report Paradigm

The Report + No-Report Paradigm was a modified version of the Report Paradigm with an embedded no-report condition. The critical adaptation for the Report + No-Report Paradigm was the addition of four central stimulus locations: above, below, left, and right of the fixation cross (Fig. 1B). Thus, the Report + No-Report Paradigm included 8 non-overlapping stimulus presentation sites between two stimulus location sets: (1) four quadrant locations and (2) four central locations. These location sets defined either report (task-relevant) or no-report (task-irrelevant) stimuli, detailed below. Moreover, the Report + No-Report Paradigm omitted the documentary background condition and only displayed the static noise background condition from the Report Paradigm because it was previously found that results did not differ between these two backgrounds^8^. And, just as in the Report Paradigm, the target stimulus was the same greyscale face that appeared for 50 ms and consisted of two sequential phases: (1) a perceptual threshold calibration and (2) testing phase.

#### Calibration phase

The aim of the calibration phase was to estimate the perceptual threshold opacity value for the target stimulus in both the quadrant and central location sets. The perceptual threshold opacity was independently defined for each location set due to differential visibility of the target stimulus nearer to the center of vision (the center locations) versus the periphery (the quadrant locations). Participants were instructed to maintain fixation to the central fixation cross at all times during the calibration phase and to immediately respond with a button press either with the index or middle finger (counterbalanced across participants) whenever they saw a target stimulus appear in any location on screen. Stimuli appeared at a jittered interval of between 1 and 1.5 s and the stimulus location for any one presentation was randomly selected among the 8 possible locations between the two location sets. The opacity values for all faces that appeared during the calibration phase were set to one of 25 pre-defined opacity values ranging between 0.01 to 0.25, incremented 0.1 over this range. Each opacity value was shown in each of the 8 locations and twice within each location. Thus, 200 stimuli per location set (quadrant and center), or 400 stimuli total were presented during the calibration phase.

Just as in the Report Paradigm, the stimulus perception responses across opacity values within each location set was modeled with a sigmoidal cumulative function from which the perceptual threshold opacity values for the centrally and peripherally located stimuli were estimated independently. The estimated perceptual threshold opacity values were used in the subsequent run of the Report + No-Report Paradigm testing phase.

#### Testing phase

The testing phase consisted of approximately 11-minute runs of 24 trials each. A trial included six sequential phases: (1) pre-stimulus, (2) first stimulus, (3) inter-stimulus, (4) second stimulus, (5) post-stimulus, and (6) response phase (Fig. 1B; Fig. S1A, B). The static noise background appeared continuous from the onset of the pre-stimulus phase through the post-stimulus phase and was then replaced with a solid gray background in the response phase. The target stimulus could appear among one of three opacity conditions: (1) no stimulus or blank (12.5%), (2) perceptual threshold (75%), and (3) fully opaque (12.5%). The addition of fully opaque stimuli allowed measurement of participant false negative detection rate. In a single trial, there could be a minimum of no target stimulus presentations (first and second stimulus phases showed blank opacity target stimuli) and a maximum of two target stimulus presentations (first and second stimulus phases showed either threshold or opaque opacity target stimuli). The frequency of the target stimulus appearance in any one of the 8 possible stimulus locations was proportional and randomly selected for each presentation. However, the stimulus could only appear once per trial within each location set. Therefore, the first stimulus was equally likely to appear in either the center or quadrant location sets, while the second stimulus was required to appear in whichever location set was not selected for the first stimulus in each trial.

The center and quadrant location sets defined task-relevant and irrelevant stimulus conditions during the testing phase. In each testing session, participants were instructed that one location set was task-relevant while the other location set was task-irrelevant and testing sessions with different location instructions were conducted on different days. For example, on a day where the center stimuli were task-relevant, they were task-relevant for all trials in the session on that day. For the stimuli that appeared in the task-relevant location set, participants were asked to recall and report via button presses on the perception of these stimuli in the trial response phase (Fig. S1A, B). Meanwhile, for stimuli that appeared in the task-irrelevant location set, participants were instructed that they would not be required to remember or respond to these stimuli. In other words, the trial response phase only inquired on the task-relevant stimuli. Therefore, the task-relevant stimuli represented a reported perceived and not perceived stimulus condition, identical to those of the Report Paradigm, while the task-irrelevant stimuli represented a no-report perceived and not perceived stimulus condition.

The pre-stimulus, inter-stimulus, and post-stimulus trial phases were jittered intervals between 6 and 10 s during which participants were instructed to maintain fixation at all times (Fig. 1B). The first and second stimulus phases consisted of a 50 ms stimulus presentation, unless there was a blank presentation when no stimulus appeared. Finally, the response phase was self-paced and presented two sequential questions for stimuli in the task-relevant location set. First, the perception question appeared (“Did you see a stimulus in a corner?” or “Did you see a stimulus near the center?” for the task-relevant quadrant and center location set conditions, respectively; Fig. S1A, B). The participants were instructed to respond with either an index and middle finger button press (counterbalanced across participants) corresponding with either a yes or no response. Next, the location question appeared (“Where was the stimulus located?”) with the numbers “1”, “2”, “3”, and “4” shown in the four locations corresponding to the task-relevant location set (Fig. S1A, B). The participants were instructed to respond with one of the four response box buttons corresponding with each of the numbered, task-relevant locations either the correct location where they had noticed a task-relevant stimulus in the current trial or to randomly guess a task-relevant stimulus location if they did not notice a stimulus in the current trial. Upon responding to the location question, the subsequent trial would begin or a post-run break screen would appear (“Great job! End of run, take a break.”). Participants did not provide overt report for task-irrelevant stimuli during the testing phase.

After completing all runs of the testing phase, participants were administered a free answer questionnaire that inquired on the general experiences during the study session, including whether if at any time during the testing phase the participants perceived stimuli in the task-irrelevant location set and how the opacity of these stimuli compared to the task-relevant stimuli.

The goal of this questionnaire was to offer a coarse assessment of what participants perceived during the testing phase, particularly for the task-irrelevant stimuli, which would later be used as the basis for a behavioral exclusion criterion for the Report + No-Report Paradigm, although this criterion resulted in no participant exclusions (For full details of all methods and materials please see the Supplementary Information).

### Functional magnetic resonance imaging (fMRI)

#### Equipment and software

fMRI measurements were acquired with 3 Tesla Siemens Magnetom scanners (Siemens, Inc.) and either a 32-channel or 64-channel head coil at the Yale Magnetic Resonance Research Center (Report Paradigm: Magnetom Trio and 32-channel head coil; Report + No-Report Paradigm: Magnetom Prisma and 64-channel head coil). A high-resolution T1-weighted, whole brain 3D structural image was acquired for each participant at each study session with a magnetization-prepared rapid gradient-epoch sequence (repetition time (TR) = 2010 ms; echo time (TE) = 2.81 mm; flip angle = 9°; field of view (FOV) = 256 × 256 mm; spatial resolution = 1 mm^3^; number of slices = 176). The blood-oxygen-dependent-level (BOLD) fMRI volumes were acquired with a multiband echo-planar imaging sequence (TR = 1000 ms; TE = 30.00 mm; flip angle = 60°; FOV = 220 × 220 mm; spatial resolution = 2 mm^3^; number of slices = 60). For the calibration phase of the Report and Report + No-Report Paradigms, each fMRI run lasted 270 and 600 s, respectively, with a corresponding total of 270 and 600 volumes of data acquired. For the testing phase of the Report and Report + No-Report Paradigms, each fMRI run lasted 720 and 700 s, respectively, with a corresponding total of 720 and 700 volumes of data acquired.

#### Overall data analysis

All fMRI analyses were completed in MATLAB (Mathworks, Inc.) using custom functions and those available through the open-source neuroimaging analysis package Statistical Parametric Mapping (SPM12; https://www.fil.ion.ucl.ac.uk/spm/software/spm12) and the SPM extension toolbox MarsBaR (http://marsbar.sourceforge.net)^60^. Whole-brain volume results and regions of interest (Figs. 3, 5, 6A–C, K; Figs. S15, S16, S19, S20C, F, I, L, S21, S22, S23; Slides 1–6^39^) are plotted on PD25-MPRAGET1w-template-500 μm.nii (0.5 mm^3^ voxels) from an MNI registration with the BigBrain dataset (https://osf.io/xkqb3/)^61,62^. Whole-brain surface results (Fig. 4) are plotted on the brain mesh image BrainMesh_ICBM152_smoothed from Surf Ice (Version 12.1; https://www.nitrc.org/projects/surfice/). For full details of all methods and materials please see the Supplementary Information.

#### Data preprocessing and artifact rejection

Standard fMRI data preprocessing was applied, including motion correction, nonlinear spatial normalization to the standard Montreal Neurological Institute (MNI) brain template space, and spatial smoothing using Gaussian kernel (FWHM = 6 mm). Additional details of preprocessing and denoising can be found in the Supplementary Information.

### Low and high-density scalp EEG (ldEEG, hdEEG)

#### Equipment and software

Non-invasive hdEEG data were collected with 257 Ag/AgCl electrodes embedded into an elastic net (Hydrocel GSN 256, Magstim Electrical Geodesics, Inc.) and recorded on a desktop computer (Power Mac G5 Quad; Mac OS X v10.5.8, Apple, Inc.) running NetStation version 4.2.2 (Magstim Electrical Geodesics, Inc.). Electrode impedance values were maintained below 50 kΩ with conductance gel (Signagel Electrode Gel, Parker Laboratories, Inc.). The EEG signal was digitized at 1000 Hz, amplified with two 128-channel amplifiers (Magstim Electrical Geodesics, Inc.) and high and low-pass hardware filtered at 0.1 and 400 Hz, respectively. Signals were acquired as Cz-referenced.

Non-invasive ldEEG were collected via 20 Ag/AgCl electrodes (Grass, Natus, Inc.) that were pasted to the participants’ scalp (Ten20 Conductive, Weaver and Company, Inc.) and arranged in the 10–20 system. Electrode leads were passed by touchproof connections into a 32-channel breakout box (XLTEK Sleep/EEG Breakout, Natus, Inc.) and recorded on clinical mobile desktop station running Natus NeuroWorks 9.2.1 Build 5186 (Natus, Inc.) Electrophysiology was sampled at 256 Hz and low-pass hardware filtered at 128 Hz. Recordings were acquired referenced to an electrode placed on the left hemisphere of the scalp in the middle of the Fz, Cz, C3, and F3 contacts and re-referenced during processing as described in the Supplementary Information.

#### Data preprocessing and artifact rejection

hdEEG and ldEEG preprocessing was implemented for each study session independently in MATLAB with an in-house, semi-automated data processing pipeline utilizing custom functions and those available by the open-source EEG processing toolbox EEGLAB^63^. EEG preprocessing consisted of two stages: (1) session-level and (2) epoch-level preprocessing. The specific steps and sequence of the preprocessing pipeline were selected from the most commonly used approaches for EEG preprocessing (e.g., see EEGLAB preprocessing and artifact rejection documentation: https://eeglab.org/tutorials). See the Supplementary Information for full preprocessing details.

### Intracranial EEG (icEEG)

#### Equipment and software

Two recording systems were used to acquire icEEG signals from 7 adult, patient participants reported in the current investigation: (1) RNS System (NeuroPace, Inc.; *N* = 6), and (2) Natus NeuroWorks Quantum (Natus, Inc.; *N* = 1). See Table S2 for individual patient participant details. RNS System icEEG recordings were sampled at 250 Hz and high and low-pass hardware filtered according to parameters set by the clinician and NeuroPace technician to optimize detection of epileptiform discharges and seizure (Table S2). The NeuroWorks Quantum icEEG recordings were acquired at a sampling rate of 4096 Hz and referenced to the first contact of a 1 × 4 frontal depth lead. Hardware low and high-pass filter frequency cutoffs were set to 0.01 and 1757 Hz, respectively. All channels and epochs were visually inspected by a trained experimenter to remove from analyses any trial with samples that included artifact (e.g., dropped signal) or epileptiform activity.

### Eye tracking and pupillometry – EyeLink

#### Equipment and software

Eye tracking and pupillometry data were collected with the EyeLink 1000 Plus System and software (version 5.09; SR Research, Inc.) running on a Dell PC desktop (Model D13M; Dell, Inc.). During hdEEG acquisition, head-fixed, binocular EyeLink recordings were acquired at 1000 Hz with a 35 mm camera lens and infrared illuminator mounted below the task LCD display. During fMRI acquisition, head-fixed (stabilized by the head-coil and padding), long range monocular (right eye) EyeLink recordings were acquired at 1000 Hz with an MR-compatible camera and infrared illuminator mounted to a mounting bar and affixed to a stand placed inside the magnet bore behind the participant.

#### Data processing

Four data types were extracted from the EyeLink recordings: (1) artifact-interpolated pupil diameter, (2) artifact-interpolated gaze position, (3) blink occurrence, and (4) microsaccade occurrence. The extraction procedure for each of these data types first involved cropping out 12 s pupil diameter and gaze position (*x* and *y*-axes) epochs for analysis centered around stimulus presentation (i.e., 6 s pre and post-stimulus presentation).

### Covert prediction of conscious perception – machine learning pipeline

A machine learning pipeline was implemented with the goal of achieving accurate trial-by-trial predictions of perceived and not perceived no-report (task-irrelevant) stimuli from the Report + No-Report Paradigm in lieu of overt report and was used to classify ISI data as “perceived” and “not perceived” to study the contribution of eye movements in the resulting hdEEG and fMRI findings. The pipeline includes trial segmentation, feature extraction, feature normalization, feature selection, information fusion, and classification stages. Full details are provided in the Supplementary Information.

### Statistical analyses

Behavioral, EyeLink, hdEEG, ldEEG, icEEG, and fMRI statistical analyses and data visualizations were executed with GraphPad Prism (version 9.1.2, GraphPad, Inc.) or custom and available MATLAB functions and toolboxes, including SPM and EEGLAB.

#### Behavioral analysis

Behavioral performance was assessed to answer two primary questions on the report, task-relevant stimuli: (1) What proportion of stimuli are seen, when stimulus opacity is at perceptual threshold, at 0% (blanks), or at 100% (fully opaque)? And, (2) what is the location accuracy for seen stimuli and for not seen stimuli? The main analyses to resolve both questions combined the performance across trials, participants, and task conditions using report (task-relevant) data from the Report and Report + No-Report Paradigms (although only the Report + No-Report Paradigm include fully opaque stimuli).

#### fMRI, hdEEG, and ldEEG Spatiotemporal Analyses

The multiple comparisons problem in statistical analyses was a concern in the current investigation because of the acquisition of high-dimensional data sets, including fMRI that is susceptible to false discoveries^64^. In addition, analyses requiring a hemodynamic response model can miss important spatiotemporal signals that do not fit the model in some brain regions^65–67^. Therefore, we implemented model-free cluster-based permutation tests to identify statistically significant spatiotemporal clusters from whole brain fMRI, hdEEG, and ldEEG. Cluster-based permutation tests have shown to be a powerful tool for limiting false positive (Type I) error rates in high-dimensional data, including EEG and fMRI.

The core principle of the cluster-based permutation analysis is to compare the experimental data to a null distribution built from a cluster-forming statistic generated after iterations of randomly permuting the experimental data^68^. A modified version of the MATLAB Mass Univariate ERP Toolbox *clust_perm1* function was implemented to generate the null distribution^69^. Full details of the cluster-based permutation analysis methods can be found in the Supplementary Information.

#### fMRI, hdEEG, ldEEG, icEEG, and eyeLink temporal analyses

Statistical analysis of all timecourse data (i.e., two-dimensional data of the structure signal by time), including fMRI k-means cluster and region of interest (ROI) timecourses, hdEEG, ldEEG, and icEEG channel timecourses, and EyeLink pupil diameter, blink rate, and microsaccade rate timecourses were implemented with an adapted version of the spatiotemporal cluster-based permutation test. The critical difference for the cluster-based permutation analysis of the timecourse data was that the cluster-forming procedure considered only temporal adjacency, whereas in the previous implementation both spatial and temporal adjacencies were used to form spatiotemporal clusters.

#### fMRI spatiotemporal conjunction and exclusive disjunction analyses

The goal of voxel-level conjunction and exclusive disjunction analyses was to emphasize the report-independent network, the report-dependent network, and the contribution of eye movement-based responses by comparing shared and unshared BOLD signals (Figs. 3E, F, 4E, F, 5E, F; Fig. S16C–F). The conjunction and exclusive disjunction voxels were queried among all whole brain gray matter voxels and over all epoch samples (20 seconds pre and post-stimulus presentation or pre and post the ISI center time).

#### fMRI anatomical clustering

Data-driven, voxel-level clustering of whole brain gray matter voxels was implemented with the clustering algorithm k-means (MATLAB *kmeans* function) on the percent change BOLD responses for report perceived minus not perceived stimuli, combined from the Report and Report + No-Report Paradigms. Voxels for anatomical clustering were selected by finding all voxels that were statistically significant for at least one sample during the first 10-second post-stimulus period, as determined from cluster-based permutation analysis of the report perceived minus not perceived stimuli percent change BOLD signal (Figs. 3A, B, 4A, B; Slide S1^39^). For the included voxels, k-means clustering was applied to the perceived minus not perceived percent change BOLD signal in the first 10 s post-stimulus averaged across participants.

#### icEEG and ldEEG latency analysis

The thalamic icEEG and scalp ldEEG ERP peak times were tested for statistically significant latencies using a peak detection procedure. For the scalp ldEEG recordings, the Oz, Pz, and Cz channels were selected for peak latency analyses because in the hdEEG data set these channels displayed the strongest early and late scalp ERPs of interest, including the N100 (75–125 ms), VAN (175–225 ms), N2 (275–325 ms), and P3 (350–650 ms). Latencies between the thalamic ERP and each scalp ERP (N100, VAN, N2, and P3) were statistically compared with two-sided Wilcoxon rank sum tests (*p* < 0.05) across participants, Holm-Bonferroni-corrected for multiple comparisons.

### Reporting summary

Further information on research design is available in the Nature Portfolio Reporting Summary linked to this article.

## Supplementary information

Supplementary Information

Reporting Summary

## Data Availability

All data generated in this study have been deposited and are available at https://bmvp.projects.nitrc.org Source data are provided with this paper.

## Source data

Source Data

## Code availability

The codes generated in this study have been deposited in Github (https://github.com) and are available at https://bmvp.projects.nitrc.org
