# Supplementary material for: Human visual consciousness involves large scale cortical and subcortical networks independent of task report and eye movement activity
Source: Nat Commun. 2022 Nov 29;13:7342. doi: 10.1038/s41467-022-35117-4 (PMC9707162; doi:10.1038/s41467-022-35117-4)
Supplement: Supplementary file 2 — Reporting Summary [file 41467_2022_35117_MOESM2_ESM.pdf]

nature portfolio

Corresponding author(s): Hal Blumenfeld

Last updated by author(s): 10/15/2022

Reporting Summary

Nature Portfolio wishes to improve the reproducibility of the work that we publish. This form provides structure for consistency and transparency in reporting. For further information on Nature Portfolio policies, see our Editorial Policies and the Editorial Policy Checklist.

Statistics

For all statistical analyses, confirm that the following items are present in the figure legend, table legend, main text, or Methods section.

n/a	Confirmed	
☐	☒	The exact sample size (*n*) for each experimental group/condition, given as a discrete number and unit of measurement
☐	☒	A statement on whether measurements were taken from distinct samples or whether the same sample was measured repeatedly
☐	☒	The statistical test(s) used AND whether they are one- or two-sided<br>*Only common tests should be described solely by name; describe more complex techniques in the Methods section.*
☒	☐	A description of all covariates tested
☐	☒	A description of any assumptions or corrections, such as tests of normality and adjustment for multiple comparisons
☐	☒	A full description of the statistical parameters including central tendency (e.g. means) or other basic estimates (e.g. regression coefficient) AND variation (e.g. standard deviation) or associated estimates of uncertainty (e.g. confidence intervals)
☐	☒	For null hypothesis testing, the test statistic (e.g. F, t, r) with confidence intervals, effect sizes, degrees of freedom and P value noted<br>*Give P values as exact values whenever suitable.*
☒	☐	For Bayesian analysis, information on the choice of priors and Markov chain Monte Carlo settings
☒	☐	For hierarchical and complex designs, identification of the appropriate level for tests and full reporting of outcomes
☒	☐	Estimates of effect sizes (e.g. Cohen's *d*, Pearson's *r*), indicating how they were calculated

Our web collection on statistics for biologists contains articles on many of the points above.

Software and code

Policy information about availability of computer code

Data collection	Custom Python scripts run on PsychoPy v1.83 for behavioral tasks
Data analysis	MATLAB R2020b; Statistical Parametric Mapping (SPM12); EEGLAB (https://eeglab.org); MarsBaR (http://marsbar.sourceforge.net); tsfresh (version 0.18.0); Stublinks (http://www.pitt.edu/~gsiegle); GraphPad Prism (version 9.1.2); custom codes generated in this study are available at https://bmvp.projects.nitrc.org

For manuscripts utilizing custom algorithms or software that are central to the research but not yet described in published literature, software must be made available to editors and reviewers. We strongly encourage code deposition in a community repository (e.g. GitHub). See the Nature Portfolio guidelines for submitting code & software for further information.

Data

Policy information about availability of data

All manuscripts must include a data availability statement. This statement should provide the following information, where applicable:

- Accession codes, unique identifiers, or web links for publicly available datasets
- A description of any restrictions on data availability
- For clinical datasets or third party data, please ensure that the statement adheres to our policy

All data generated in this study have been deposited and are available at https://bmvp.projects.nitrc.org

Field-specific reporting

Please select the one below that is the best fit for your research. If you are not sure, read the appropriate sections before making your selection.

☒ Life sciences ☐ Behavioural & social sciences ☐ Ecological, evolutionary & environmental sciences

For a reference copy of the document with all sections, see nature.com/documents/nr-reporting-summary-flat.pdf

Life sciences study design

All studies must disclose on these points even when the disclosure is negative.

Sample size	Sample sizes were determined from pilot data sets used to predict the yield of trials in our final data set. The total number of subjects recruited in this study (approximately 90 healthy MRI participants and 120 healthy EEG participants) exceed by at least double the sample sizes typically reported in the literature for similar studies (e.g., see Szucs and Ioannidis, NeuroImage, 2020). In the case of patient participants, the sample size was determined by the availability of this rare patient population.
Data exclusions	There were multiple levels of data exclusion (e.g., trial, task block, session, and participant-level), including based on behavioral performance, movement in the MRI, eye blink events, and insufficient data for analysis. All data exclusions are fully detailed in the Supplementary Information.
Replication	For both the MRI and EEG procedures, two independent subject groups were recruited (with the exception of four participants present in both groups). Results from each group were independently analyzed and found to replicate findings. Final analyses combined between the acquired MRI and EEG data sets.
Randomization	Participants were allocated to experimental groups randomly and all study conditions were counterbalanced, including response buttons and task sequence. Full details on randomization are described in the Supplementary Information.
Blinding	The experimenter was not blinded to the participant conditions. Instructions to the participant depended on the experimenter knowing the task condition.

Reporting for specific materials, systems and methods

We require information from authors about some types of materials, experimental systems and methods used in many studies. Here, indicate whether each material, system or method listed is relevant to your study. If you are not sure if a list item applies to your research, read the appropriate section before selecting a response.

Materials & experimental systems

n/a	Involved in the study	
☒	☐	Antibodies
☒	☐	Eukaryotic cell lines
☒	☐	Palaeontology and archaeology
☒	☐	Animals and other organisms
☐	☒	Human research participants
☒	☐	Clinical data
☒	☐	Dual use research of concern

Methods

n/a	Involved in the study	
☒	☐	ChIP-seq
☒	☐	Flow cytometry
☐	☒	MRI-based neuroimaging

Human research participants

Policy information about studies involving human research participants

Population characteristics	There were four primary data sets gathered from the healthy participants: (1) Report Paradigm (detailed in the Visual Perception Paradigms section) with simultaneous fMRI recording (N = 37; mean age = 27.22 years; age range = 18-42 years; females = 17; right-handed = 35), (2) Report Paradigm with simultaneous hdEEG and binocular eye tracking and pupillometry recordings (N = 59; mean age = 26.20 years; age range = 19-43 years; females = 37; right-handed = 53), (3) Report + No-Report Paradigm (detailed in the Visual Perception Paradigms section) with simultaneous fMRI and monocular eye tracking and pupillometry recordings (N = 65; mean age = 24.77 years; age range = 18-46 years; females = 39; right-handed = 64), and (4) Report + No-Report Paradigm with simultaneous hdEEG and binocular eye tracking and pupillometry recordings (N = 65; mean age = 24.58 years; age range = 18-46 years; females = 39; right-handed = 63). Two primary data sets were gathered from the patient participants: (1) Report Paradigm with simultaneous low-density scalp EEG (ldEEG) and thalamic intracranial EEG (icEEG) (N = 6; mean age = 24.17 years; age range = 20-31; females = 4; right-handed = 6) and (2) Report Paradigm with icEEG alone, without concurrent ldEEG (N = 1; age = 29; female = 1; left-handed = 1). Full details on participants are reported in the Supplementary Information.
Recruitment	Healthy participants were recruited by flyer and online advertisements to the Yale/New Haven, CT community. Patient participants were recruited by referral from their primary physician. Self-selection biases are a potential limitation of the

recruitment strategy, although a male-female balance was maintained across subject pools. Self-selection biases are unlikely to introduce a meaningful impact on the results reported in the current investigation.

Ethics oversight: The study procedures were approved by the Yale University and University of Pittsburgh Institutional Review Boards.

Note that full information on the approval of the study protocol must also be provided in the manuscript.

Magnetic resonance imaging

Experimental design

Design type: Event-related design

Design specifications: The behavioral task was broken into either four or five 10 to 12-minute blocks of 32 or 24 trials each, respectively. Full details on task design and sequence are reported in the Supplementary Information.

Behavioral performance measures: Participant button press responses to perception and location validation questions were recorded. The average perception rate and location accuracy across all session trial subtypes was used to assess performance. Full details on behavioral performance is reported in the Supplementary Information.

Acquisition

Imaging type(s): Functional

Field strength: 3T

Sequence & imaging parameters: fMRI measurements were acquired with 3 Tesla Siemens Magnetom scanners (Siemens, Inc.) and either a 32-channel or 64-channel head coil at the Yale Magnetic Resonance Research Center (Report Paradigm: Magnetom Trio and 32-channel head coil; Report + No-Report Paradigm: Magnetom Prisma and 64-channel head coil). A high-resolution T1-weighted, whole brain 3D structural image was acquired for each participant at each study session with a magnetization-prepared rapid gradient-epoch sequence (repetition time (TR) = 2010ms; echo time (TE) = 2.81mm; flip angle = 9 degrees; field of view (FOV) = 256x256mm; spatial resolution = 1mm3; number of slices = 176). The blood-oxygen-dependent-level (BOLD) fMRI volumes were acquired with a multiband echo-planar imaging sequence (TR = 1000ms; TE = 30.00mm; flip angle = 60 degrees; FOV = 220x220mm; spatial resolution = 2mm3; number of slices = 60).

Area of acquisition: Whole brain

Diffusion MRI: ☐ Used ☒ Not used

Preprocessing

Preprocessing software: SPM12

Normalization: Subject structural and functional images were normalized via non-linear transformation.

Normalization template: ICBM152

Noise and artifact removal: Preprocessed BOLD volumes were passed through a 5-staged denoising procedure previously published from our group. In sequence, (1) volumes were grey matter masked (i.e., excluding non-grey matter voxels), (2) application of 128Hz high-pass filter, (3) removal of head motion-related signals by regression utilizing a general linear model with the six rigid-body motion parameters estimated during preprocessing image realignment, (4) rejection of voxels at timepoints with a BOLD signal volume-to-volume root mean squared difference that exceeded 5 (DVARS)6, and (5) rejection of voxels at timepoints that exceeded a framewise displacement (FD) threshold of 0.3 (FD was calculated as the sum of the absolute values of change in head movement among the six rigid-body motion parameters). Rejected voxels and timepoints were replaced with the MATLAB designation of not-a-number or “NaN”.

Volume censoring: Censoring was completed on the voxel-level, not volume-level. See "Noise and artifact removal" above.

Statistical modeling & inference

Model type and settings: Model-free analyses were implemented. Full details on MRI statistical analyses are reported in the Supplementary Information.

Effect(s) tested: Model-free analyses were implemented. Full details on MRI statistical analyses are reported in the Supplementary Information.

Specify type of analysis: ☒ Whole brain ☐ ROI-based ☐ Both

Statistic type for inference (See Eklund et al. 2016): Model-free analyses were implemented. Full details on MRI statistical analyses are reported in the Supplementary Information.

Correction: Cluster-based permutation analysis. Full details on MRI statistical analyses are reported in the Supplementary Information.

Models & analysis

n/a	Involved in the study
☒	☐ Functional and/or effective connectivity
☒	☐ Graph analysis
☒	☐ Multivariate modeling or predictive analysis
